# Age-specific cut-off levels of anti-Müllerian hormone can be used as diagnostic markers for polycystic ovary syndrome

**DOI:** 10.1186/s12958-021-00755-8

**Published:** 2021-05-22

**Authors:** Fahimeh Ramezani Tehrani, Maryam Rahmati, Fatemeh Mahboobifard, Faezeh Firouzi, Nazanin Hashemi, Fereidoun Azizi

**Affiliations:** 1 Reproductive Endocrinology Research Center, Research Institute for Endocrine Sciences, Shahid Beheshti University of Medical Sciences, P.O. Box:19395-4763, 24 Parvaneh, Yaman Street, Velenjak, I.R 1985717413 Tehran, Iran; 2 Endocrine Research Center, Research Institute for Endocrine Sciences, Shahid Beheshti University of Medical Sciences, Tehran, Iran

**Keywords:** Anti-Müllerian hormone, Polycystic ovary syndrome, Age-specific, Diagnosis, Bayesian method

## Abstract

**Background:**

The majority of available studies on the AMH thresholds were not age-specific and performed the receiver operating characteristic curve (ROC) analysis, based on variations in sensitivity and specificity rather than positive and negative predictive values (PPV and NPV, respectively), which are more clinically applicable. Moreover, all of these studies used a pre-specified age categorization to report the age-specific cut-off values of AMH.

**Methods:**

A total of 803 women, including 303 PCOS patients and 500 eumenorrheic non-hirsute control women, were enrolled in the present study. The PCOS group included PCOS women, aged 20–40 years, who were referred to the Reproductive Endocrinology Research Center, Tehran, Iran. The Rotterdam consensus criteria were used for diagnosis of PCOS. The control group was selected among women, aged 20–40 years, who participated in Tehran Lipid and Glucose cohort Study (TLGS). Generalized additive models (GAMs) were used to identify the optimal cut-off points for various age categories. The cut-off levels of AMH in different age categories were estimated, using the Bayesian method.

**Main results and the role of chance:**

Two optimal cut-off levels of AMH (ng/ml) were identified at the age of 27 and 35 years, based on GAMs. The cut-off levels for the prediction of PCOS in the age categories of 20–27, 27–35, and 35–40 years were 5.7 (95 % CI: 5.48–6.19), 4.55 (95 % CI: 4.52–4.64), and 3.72 (95 % CI: 3.55–3.80), respectively. Based on the Bayesian method, the PPV and NPV of these cut-off levels were as follows: PPV = 0.98 (95 % CI: 0.96–0.99) and NPV = 0.40 (95 % CI: 0.30–0.51) for the age group of 20–27 years; PPV = 0.96 (95 % CI: 0.91–0.99) and NPV = 0.82 (95 % CI: 0.78–0.86) for the age group of 27–35 years; and PPV = 0.86 (95 % CI: 0.80–0.94) and NPV = 0.96 (95 % CI: 0.93–0.98) for the age group of 35–40 years.

**Conclusions:**

Application of age-specific cut-off levels of AMH, according to the GAMs and Bayesian method, could elegantly assess the value of AMH in discriminating PCOS patients in all age categories.

**Supplementary Information:**

The online version contains supplementary material available at 10.1186/s12958-021-00755-8.

## Background

 Polycystic ovary syndrome (PCOS) is the most common endocrine disorder in reproductive women. The prevalence of PCOS ranges from 5 to 20 % in different studies, depending on the recruitment process of the study population, the criteria used for its definition, and the method used to define each criterion [1]. According to the Rotterdam criteria, which were first introduced in 2004, diagnosis of PCOS requires the presence of at least two of the following findings: (i) oligoanovulation (OA); (ii) clinical or biochemical hyperandrogenism (HA); and (iii) polycystic ovarian morphology (PCOM), based on the ultrasound findings. Also, other etiologies for excess androgen production must be excluded [2]. However, the inclusion of PCOM in the definition of PCOS has caused major concerns regarding the validity and reliability of its assessment and highlighted the need for revising its definition, given the application of advanced high-resolution ultrasound devices [3].

Several efforts have been made to introduce a more reliable alternative for PCOM. The anti-Müllerian hormone (AMH), given its exclusive production by granulosa cells of the ovary, has been shown to be the best marker, reflecting the antral follicle count (AFC) [4]. Researchers have also attempted to identify the optimal diagnostic threshold for AMH to precisely identify PCOM [5] for the PCOS criteria. However, there is no universally accepted threshold [5]. Besides AFC, AMH is correlated with HA [6–8], oligomenorrhea, and menstrual disorders [9].

Several studies have suggested that AMH could be used as a surrogate marker for the diagnosis of PCOS [10–12]. However, the majority of these studies have some shortcomings, mainly due to not being age-specific, as marked changes occur in AMH across the reproductive lifespan in the normal population, and there is the possibility of AMH decline in a less rapid manner in women with PCOS [13, 14]. Therefore, in the present study, we aimed to introduce the age-specific cut-off levels of AMH for the prediction of PCOS, using a Bayesian method, and to identify the optimal cut-off points for various age categories, using generalized additive models (GAMs).

## Methods

This study was conducted on 839 women at reproductive age, including 321 PCOS patients (case group) and 518 eumenorrheic non-hirsute women (control group), aged 20–40 years. The controls were selected among the participants of Tehran Lipid and Glucose cohort Study (TLGS), which is a cohort study among a representative sample of residents in Tehran, Iran. Face-to-face interviews were conducted with 1060 women, aged 18–45 years. The reproductive history, with emphasis on the menstrual cycle, gynecological history, hyperandrogenic symptoms, and family history of irregular menstrual cycle and hirsutism were collected. Hirsutism was assessed by a trained general practitioner, under the supervision of a single gynecologist, using the modified Ferriman-Gallwey (mFG) scoring method. For cases with acne and/or an initial mFG score > 3 and/or a menstrual disorder (defined as a menstrual interval < 21 days or > 35 days), the androgen and/or progesterone profile in the mid-luteal phase was assessed. The details are published elsewhere [15]. To select the controls, we excluded women with at least one of the PCOS criteria, including irregular or unpredictable menstrual cycles, subclinical anovulation (progesterone level < 4 ng/mL in two consecutive cycles), and biochemical and/or clinical HA (*n* = 307). In addition, the exclusion criteria were as follows: (1) menopause (*n* = 22); (2) history of hysterectomy, oophorectomy, or ovarian surgeries (*n* = 36); (3) history of endocrine disorders or use of medications that could affect the function of the hypothalamic-pituitary-gonadal (HPG) axis (*n* = 34); (4) lack of available information on the reproductive history (*n* = 60); (5) being in the age range of < 20 or > 40 years (*n* = 83); and (6) having an outlier AMH value (*n* = 18).

The PCOS group consisted of PCOS women, aged 20–40 years, who were referred to the Reproductive Endocrinology Research Center, Tehran, Iran. The Rotterdam criteria were used for diagnosis of PCOS, based on the presence of at least two of the three following findings: (i) OA; (ii) HA; and (iii) PCOM on ultrasound. OA was considered as vaginal bleeding episodes at no less than 35-day intervals or progesterone levels < 4 ng/mL in two consecutive cycles. Also, HA was defined as clinical hyperandrogenism, that is, the presence of hirsutism (mFG ≥ 8), acne, or androgenic alopecia and/or biochemical hyperandrogenism (BH).

BH was detected based on the free androgen index (FAI) and the level of dehydroepiandrosterone sulfate (DHEAS) and/or androstenedione (A4) above the upper 95th percentile in women (*n* = 362), who were not using any hormonal medications and had no clinical evidence of HA, OA, or PCOM. Specifically, the upper normal limits were as follows: DHEAS = 246 µg/dL, FAI = 5.47, total testosterone = 0.88 ng/mL, A4 = 2.3 ng/mL [16]. PCOM was diagnosed, based on the presence of ≥ 12 follicles 2–9 mm in each ovary and/or increased ovarian volume (10 cm^3^). Out of 321 women with PCOS, those with an outlier AMH value were excluded (*n* = 18).

An overnight fasting venous blood sample was obtained from each participant on the second or third day of their spontaneous or progesterone-induced menstrual cycle. The samples were centrifuged within 30–45 min of collection and stored at -80 °C until further analysis. All AMH measurements were performed by an experienced laboratory technician in the same laboratory at the Research Institute for Endocrine Sciences, using the same assay method and kit (Gen II Kit, Beckman Coulter, Inc., Fullerton, California, USA), as well as the same Sunrise ELISA reader (Tecan Co., Salzburg, Austria). Also, the AMH Gen II control (A79766) was used at two concentrations to monitor the accuracy of the assays. The intra- and inter-assay coefficients of variation were 1.9 and 2.0 %, respectively.

### Statistical analysis

Data are presented as mean ± standard deviation (SD) for numerical variables with normal distributions, as median and interquartile range (IQR) for variables with skewed distributions, and as number (percentage) for categorical variables. The characteristics of women with PCOS and the controls were compared, using student’s t-test for normally distributed continuous variables and Chi-square test for categorical data. Mann–Whitney U test was also performed to compare variables with skewed distributions.

To measure the outliers of AMH, Tukey’s test was performed, as it makes no distributional assumptions. Accordingly, all values, which were more than 1.5 times the IQR below the first quartile or above the third quartile (either below Q1 − 1.5 IQR or above Q3 + 1.5 IQR), were excluded. To detect the correlation of AMH with age and body mass index (BMI), a scatter plot matrix was drawn, and a fractional polynomial (FP) regression model was fitted to identify the non-linear relationship between continuous variables (age and AMH level). Pearson’s or Spearman’s correlation test was also used to examine the correlation of AMH with age and BMI.

It seems that subjective categorization of age (i.e., 20–25, 25–30, 30–35, and 35–40 years) is not appropriate for age-specific predictions; therefore, GAMs were used to identify the cut-off points for age, considering the association of this variable with AMH [17]. These models consider non-parametric functions of predictive variables (i.e., age and AMH), which are associated with the dependent variable (i.e., PCOS) using a logit link function and provide the optimal cut-off points, based on the area under the ROC curve (AUC) maximization [18].

Next, the cut-off values of AMH were estimated, based on the negative and positive predictive values (NPV and PPV, respectively) for different age categories, using the Bayesian method, introduced by Vradi et al. [19]. Generally, this method models the probability of PCOS with a step function, based on the predictive values, and estimates the cut-off value, as well as the predictive values. Here, the cut-off value was a parameter of the model, and therefore, a Bayesian method could be applied. This method allows direct probability interpretations of parameters, based on the observed data and uses both prior and sample information [20]. Moreover, the predictive summary index (PSI) was applied, which is a frequentist approach to determine the optimal overall and age-specific cut-off levels of AMH. It estimates the optimal cut-off value by maximizing the difference in predictive values for all possible cut-off points and is expressed as follows [19]:

$$PSI={max}_{cutoff}\left\{{PPV}_{cutoff}+{NPV}_{cutoff}-1\right\}$$

For the latter approach, the confidence intervals were calculated by the bootstrap method.

There was not an adequate sample size to calculate the age-specific cut-off values of AMH for different PCOS phenotypes; however, these cut-offs for different phenotypes (regardless of age) were estimated, using the proposed Bayesian method.

Data were analyzed using OpenBUGS version 3.2.3 and R software version 3.6.1. The optimal cut-off points for age were obtained by the CatPredi package in R software. *P*-value less than 0.05 was considered statistically significant.

## Results

Of a total of 839 subjects, aged 20–40 years, 321 (38.2 %) women had PCOS, and 518 (61.8 %) were normo-ovulatory. The participants with outlier AMH levels were detected and excluded, based on the boxplot method. Finally, 803 subjects, including 303 (37.7 %) patients with PCOS and 500 (62.3 %) normo-ovulatory women, were examined. Women in the PCOS group were younger (27.9±4.6 vs. 33.1±4.6) than the control group and had significantly higher AMH levels (6.21, IQR: 3.90–9.03 vs. 1.70, IQR: 0.85–2.81; *P* < 0.001). There was no significant difference in terms of BMI between the PCOS and control groups (*P* = 0.5). The clinical and endocrine characteristics of the PCOS group and normo-ovulatory controls are summarized in Table 1.

**Table 1:** Clinical and endocrine characteristics of the PCOS cases and normo-ovulatory controls

Variable	Normo-ovulatory (*n* = 500)	PCOS (303)	*p*-value
Age (years)	33.1 ± 4.6	27.9 ± 4.6	**< 0.001**^**a**^
Age at menarche (years)	13.5 ± 1.4	13.1 ± 1.6	**0.002**^**a**^
Number of pregnancy	2.5 ± 1.2	0.4 ± 0.8	**< 0.001**^**a**^
Number of delivery	2.1 ± 0.9	0.3 ± 0.6	**< 0.001**^**a**^
Number of abortion	1.2 ± 0.6	0.1 ± 0.5	**< 0.001**^**a**^
BMI (kg/m^2^)	26.5 ± 4.5	26.7 ± 5.7	0.5
WC (cm)	83.5 ± 10.3	85.5 ± 12.8	**0.01**^**a**^
Wrist (cm)	15.7 ± 0.9	15.3 ± 1.2	**< 0.001**^**a**^
Hip (cm)	103.2 ± 8.8	104.4 ± 10.6	0.08
WHR	0.8 ± 0.1	0.82 ± 0.1	0.9
WHtR	0.5 ± 0.1	0.5 ± 0.1	0.8
AMH (ng/ml) ^b^	1.70 (0.85–2.81)	6.21 (3.90–9.03)	**< 0.001**^**a**^

Note: Data are presented as mean ± standard deviation unless otherwise indicated. Independent t-test, or Mann-Whitney test were used as appropriate

*BMI *body mass index, *AMH *anti-mullerian hormone, *WC *waist circumference, *WHR *waist-to-hip ratio, *WHtR *waist-to-height ratio

^a^Statistically significant result (*p* < 0.05)

^b^ Median (IQR 25-75 %)

**Fig. 1:**
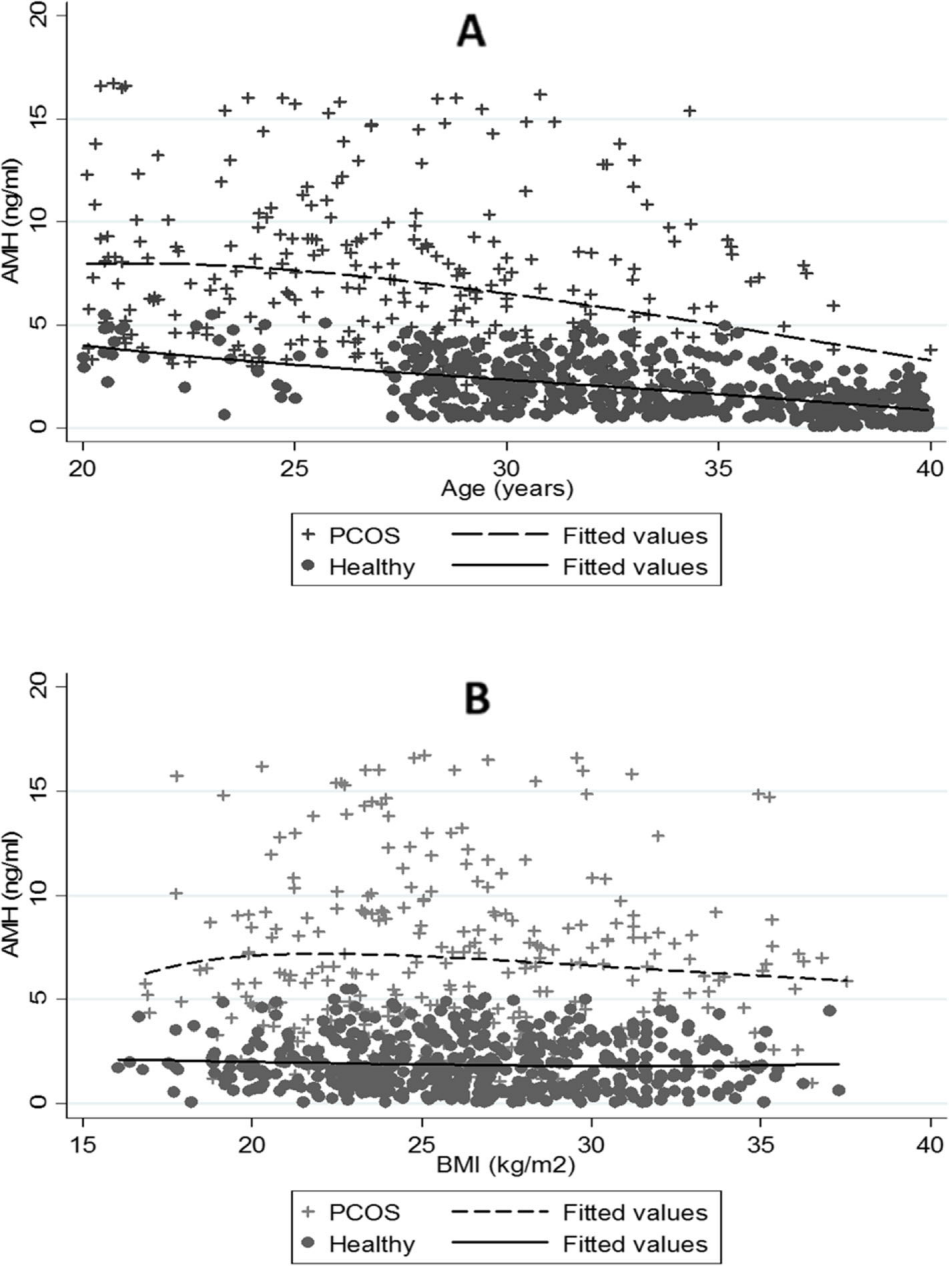
Scatter plot matrix of AMH levels versus (**a**) age and (**b**) BMI

**Fig. 2:**
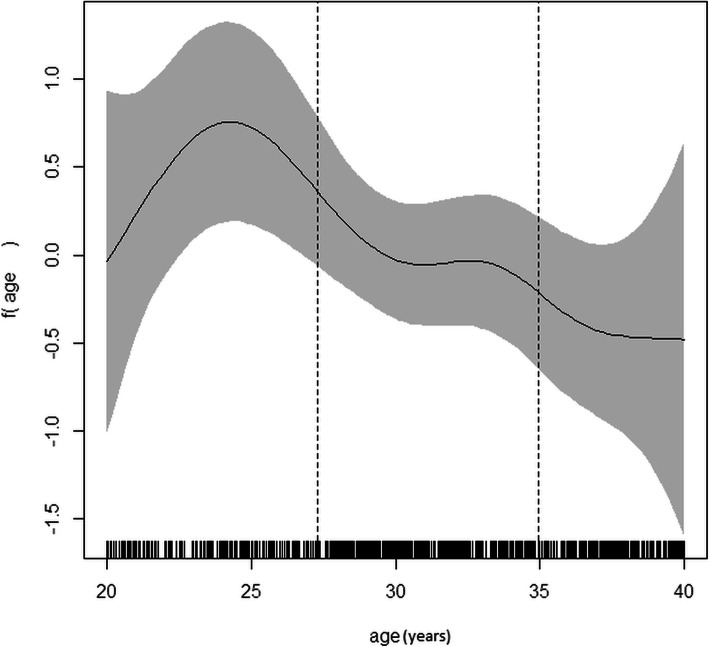
The optimal location of cut points for age variable based on the generalized additive models (GAMs)

**Fig. 3:**
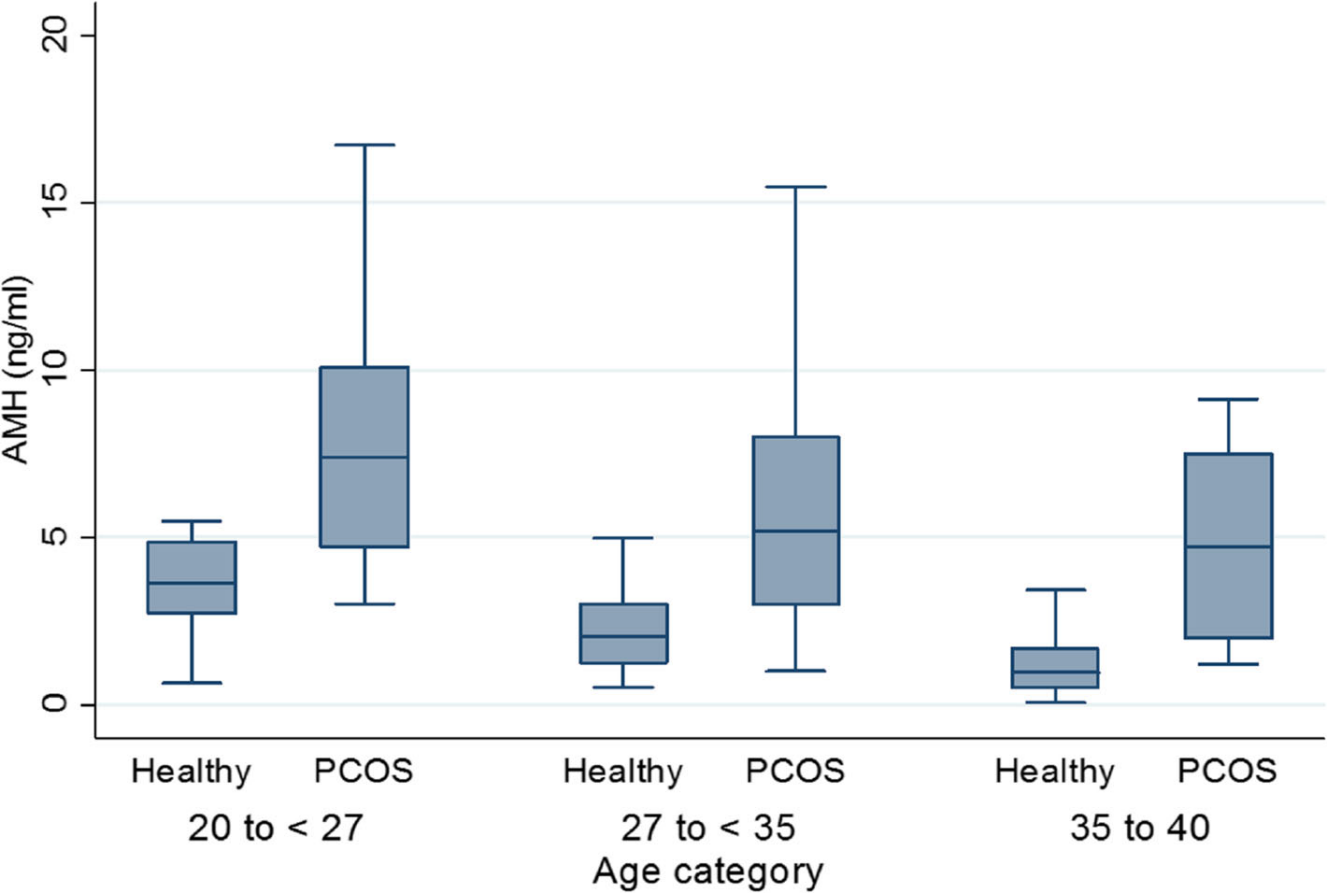
Box plots showing the values of serum AMH in ng/ml within controls and PCOS and with stratification by age group

As shown in Fig. 1, the scatter plot matrix indicated that AMH levels decreased with increasing age in both PCOS and normo-ovulatory groups (r_total_ = -0.63, *P* < 0.001); however, there was no significant association between the AMH level and BMI (r_total_ = -0.05, *P* = 0.12). The cut-off points for age were obtained, based on the GAM method, where the predictive variables (i.e., age and AMH) were included in the logit link function. Two optimal cut-off points were identified at the age of 27 and 35 years, and the age groups were classified as follows: 20–27, 27–35, and 35–40 years (Fig. 2). The distribution of AMH in the PCOS and control groups, based on age stratification, is presented in Fig. 3. The AMH values were compared between the PCOS and normo-ovulatory control groups in an age-stratified analysis. The level of AMH decreased with increasing age, and a significant difference was found between the PCOS and normo-ovulatory groups (*P* < 0.001) (Table 2).

**Table 2:** Median and (IQR 25–75 %) for AMH (ng/ml) variable by age categories in PCOS and normo-ovulatory

Age category^a^	Normo-ovulatory (*n* = 500)	PCOS (*n* = 303)	*p*-value
**20 to < 27**	*N* = 30	*N* = 134	< 0.001
	3.6 (2.60–4.85)	7.4 (4.70–10.12)	
**27 to < 35**	*N* = 284	*N* = 151	< 0.001
	2.03 (1.23–3.02)	5.16 (2.97–8.00)	
**35 to 40**	*N* = 186	*N* = 18	< 0.001
	0.97 (0.48–1.68)	4.72 (1.89–7.60)	

^a^Age categories were determined using the generalized additive models (GAMs)

**Fig. 4:**
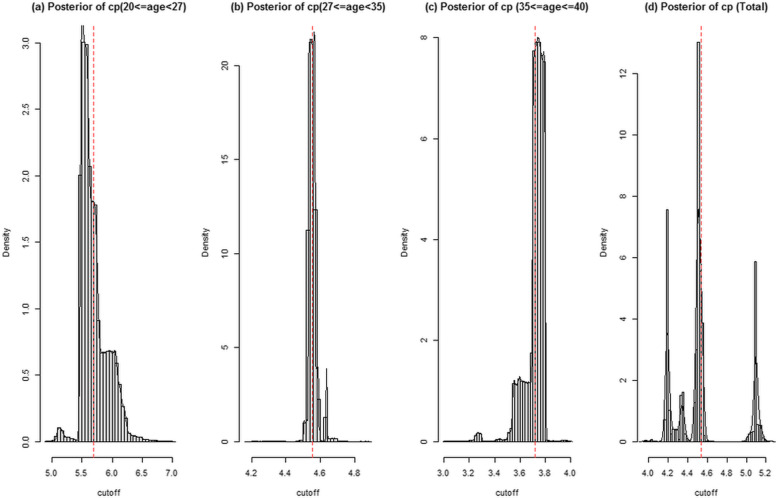
Plot of the posterior distribution for the parameter cut-off of AMH variable in (**a**) 20 < = age < 27, (**b**) 27 < = age < 35, (**c**) 35 < = age < = 40, and (**d**) total participants estimated by the Bayesian model. The vertical lines denote the median of the distribution

Figure 4 presents the posterior distribution of the cut-off values of AMH for PCOS diagnosis in the aforementioned age categories and the total population. The posterior mean of the cut-off AMH values in the age groups was as follows: 5.7 (95 % CI: 5.48–6.19), 4.55 (95 % CI: 4.52–4.64), and 3.72 (95 % CI: 3.55–3.80) for the age groups of 20–27, 27–35, and 35–40 years, respectively. At these cut-off levels, the Bayesian posterior mean values, based on PPV and NPV, were as follows: PPV = 0.98 (95 % CI: 0.96–0.99) and NPV = 0.40 (95 % CI: 0.30–0.51) for the age group of 20–27 years; PPV = 0.96 (95 % CI: 0.91–0.99) and NPV = 0.82 (95 % CI: 0.78–0.86) for the age group of 27–35 years; and PPV = 0.86 (95 % CI: 0.80–0.94) and NPV = 0.96 (95 % CI: 0.93–0.98) for the age group of 35–40 years (Table 3). Regardless of the age categories, the posterior mean of the cut-off AMH value was 4.54 (95 % CI: 4.18–5.12), with PPV of 0.93 (95 % CI: 0.89–0.99) and NPV of 0.83 (95 % CI: 0.79–0.87). The boxplot and the cut-off values of AMH within PCOS phenotypes, (regardless of age specification due to the lack of enough sample size), are presented in supplementary Fig. 1 and supplementary Table 1. Overall, the posterior means of the cut-off AMH values for the PCOS phenotypes were in a similar range.

**Table 3:** Cut-off, PPV and NPV for AMH values (ng/ml), based on Bayesian approach and PSI method with 95% CI

	Age category^a^	Estimated cutoff	PPV	NPV
Bayesian method	20 to < 27	5.70 (5.48, 6.19)	0.98 (0.96,0.99)	0.40 (0.30,0.51)
	27 to < 35	4.55 (4.52,4.64)	0.96 (0.91,0.99)	0.82 (0.78,0.86)
	35 to 40	3.72 (3.55,3.80)	0.86 (0.80,0.94)	0.96 (0.93,0.98)
	Total	4.54 (4.18, 5.12)	0.93 (0.89, 0.99)	0.83 (0.79, 0.87)
PSI method	20 to < 27	8.51 (7.12, 9.38)	0.85 (0.81, 0.89)	0.48 (0.25,0.72)
	27 to < 35	2.91 (2.11, 3.72)	0.65 (0.61,0.69)	0.92 (0.89,0.95)
	35 to 40	1.88 (1.25, 2.21)	0.57 (0.57,0.61)	0.94 (0.91,0.97) 0.83 (0.79,0.86)
	Total	4.60 (4.13, 5.54)	0.93 (0.89,0.96)	

Note: *PPV *Positive predictive value, *NPV *Negative predictive value, *PSI *predictive summary index, *CI *confidence interval

^a^Age categories were determined using the generalized additive models (GAMs)

The optimal overall and age-specific cut-off values for AMH, based on the PSI method, are presented in Table 3. The cut-off AMH value in the total population was 4.60 (95 % CI: 4.13–5.54), with PPV of 0.93 (95 % CI: 0.89–0.96) and NPV of 0.83 (95 % CI: 0.79–0.86). Also, the cut-off values of AMH for the age categories of 20–27, 27–35, and 35–40 years were 8.51 (95 % CI: 7.12–9.38), 2.91 (95 % CI: 2.11–3.72), and 1.88 (95 % CI: 1.25–2.21), respectively.

We also considered several simulation scenarios with different sample sizes to compare two methods (Bayesian and PSI). In addition, three different prior specifications, including uninformative, informative and mixture priors were considered. The results showed that the Bayesian method gives much better coverage than the PSI method for a small sample size. Also, for all considered priors, the resulting estimators are on average unbiased. For the PSI method in a small sample size (under 50), the bias of the estimate of the cut point is far too high in absolute terms. The length of the credible interval for the Bayesian method was always narrower compared to PSI (data not shown).

## Discussion

In the present study, which was conducted on a large population of women diagnosed with PCOS (based on the Rotterdam criteria) and eumenorrheic non-hirsute controls, we reported age-specific cut-off values of AMH for the prediction of PCOS, using the Bayesian method. This method is based on reporting PPV and NPV, which demonstrate more clinical utility for clinicians, compared to the ROC analysis, indicating sensitivity and specificity [19]. Additionally, this method has the advantage of dealing with a small sample size because of not relying on asymptotic theory [21, 22]. A comparison of the Bayesian method with the PSI index (as an alternative frequentist method) in our data set, revealed that the Bayesian method provides more informative results. Moreover, we categorized age, based on GAM models, which provide more reliable cut-off points than subjective categorization of age. Based on these methods, an AMH cut-off value of 5.7 ng/mL was obtained for women, aged 20–27 years, with PPV of 0.98 (95 % CI: 0.96–0.99). The AMH cut-off value was 4.55 ng/mL for women aged 27–35 years and 3.72 for those aged 35–40 years, with PPVs of 0.96 (95 % CI: 0.91–0.99) and 0.86 (95 % CI: 0.80–0.94), respectively.

Considering the limitations of the Rotterdam criteria, including ultrasound challenges (e.g., lack of universally approved cut-off points for exploring the follicle count to define PCOM, limited availability of ultrasound devices, and high cost) [5] and regional variations in the clinical presentations of HA [23, 24], use of a single assay to diagnose PCOS has been encouraged. Among candidate markers, AMH seems to be an eligible candidate. The circulating level of AMH is not majorly influenced by the menstrual cycle or use of contraceptives [25]. It also shows a strong association with AFC and serves as a reliable marker for the ovarian reserve [26].

AMH has been shown to be a proper alternative for the follicle count on ultrasound images in the Rotterdam criteria [10, 27], considering its superior AUC and higher sensitivity and specificity than the follicle count [3]. This marker can also discriminate between PCOM and PCOS [28, 29, 5, 30]. AMH has been proposed as an effective endocrine factor in the pathophysiology of PCOS, considering its elevated serum level in PCOS, its overproduction by granulosa cells in anovulatory PCOS, its cross-communication with luteinizing hormone (LH) and follicle-stimulating hormone (FSH) leading to HA, and finally, its correlation with the severity of ovulatory dysfunction and PCOS [28, 31].

It is well established that follicle count per ovary and ovarian volume show decreasing trends with age. The use of these markers, by establishing age-specific thresholds, has provided higher sensitivity and specificity for diagnosis of PCOS, compared to a single threshold [32]. Moreover, studies have shown that AMH levels gradually decrease with aging [13]; however, the rate of AMH decline may not be the same for all women at reproductive age [14]. It has been shown that the ovarian pool is depleted in a more gradual manner in the ovaries of PCOS women, compared to non-PCOS women [33].

 Evidence shows that the AMH reduction rate accelerates after the age of 40 years [34]. However, this decline has been found to be slower over time in PCOS women than normo-ovulatory women, indicating sustained fertility in PCOS women [33]. Several efforts have been made to provide an optimal single threshold of AMH for the precise diagnosis of PCOS. According to a meta-analysis of ten studies, as well as a study utilizing three main criteria (NIH, Rotterdam, and AE-PCOS criteria) to distinguish PCOS [35, 36], the AMH cut-off value of ~ 4.7 ng/mL was the best single threshold to distinguish PCOS women, based on the Rotterdam criteria. However, the majority of these studies did not report age-specific thresholds [35] and used ROC analysis, which represents sensitivity and specificity rather than PPV and NPV.

In the present study, we used the Bayesian approach [19]. Besides providing PPV and NPV values, this method allows us to consider a posterior probability distribution, according to previously reported thresholds to improve the estimations. Moreover, age categories have been subjectively identified in all previous studies, while we used GAMs with a logit link function to obtain the optimal cut-off points for age, based on AUC maximization [18, 37]. Generally, GAMs consider the nonlinear dependence between the covariates and the expected value of the response variable and provide more flexibility than generalized linear models by considering the non-parametric functions of predictive variables [17].

Besides the Bayesian method, we also reported the optimal overall and age-specific cut-off values for AMH with an alternative approach, that is, PSI. Similar to the Bayesian method, PSI is based on reporting PPV and NPV. However, it does not directly provide confidence intervals for the parameters of interest. PSI is indirectly calculated, using bootstrap samples. In the present study, the optimal single thresholds of AMH, calculated with both Bayesian and PSI methods, were 4.54 and 4.60, respectively. These thresholds could noticeably discriminate PCOS women (PPV = 0.93) from non-PCOS women (NPV = 0.83). After stratifying the AMH values by age, it was found that PPV for the younger age categories (20–27 and 27–35 years) and NPV for the oldest age category (35–40 years) were improved. Overall, the PSI approach showed wider confidence intervals and lower PPVs, compared to the Bayesian method.

There is no consistence regarding the association between BMI and PCOS or between BMI and AMH [36, 38, 39]. In this regard, a meta-analysis conducted by Moslehi et al. [40] revealed that markers of ovarian reserve (AMH and FSH) were significantly lower in obese women, compared to non-obese women. Also, BMI was negatively correlated with AMH in all populations and with FSH in fertile non-PCOS subgroups [40]. It seems that the association between AMH and PCOS is affected by BMI [31, 38]. In the present study, there was no significant association between AMH and BMI in either of the groups, and no significant difference was found in terms of BMI between PCOS and control women. Therefore, we did not adjust the age-specific AMH cut-off values for BMI.

The present study, which included community controls, benefitted from the Bayesian method to determine the cut-off AMH values for different age groups, identified by GAMs. This method provides PPVs and NPVs, which are more useful for clinical decision-making. Moreover despite the limitations of the small sample size of 30 cases in controls (20–27 years) and 18 in PCOS (35–40 years), the Bayesian method was very tractable in estimating the parameters of interest, resulting in point estimators (e.g. posterior mean) that are practically unbiased in all scenarios, for all prior constellations and sample size assumptions; this issue is in line with recommendations to use Bayesian over frequentist estimation in small sample sizes [41, 42]. Also, the intra-assay and inter-assay variabilities of AMH measurements seem to be ignored, since all AMH assays were performed by an expert in the same laboratory. Moreover, the participants in the current study were in wider age groups, compared to previous studies; this enabled us to provide thresholds for all reproductive age groups. On the other hand, our study had some limitations. First, due to the decline in PCOS clinical manifestations, concerns and consequences over the lifespan [43–45], the PCOS population is significantly younger than control subjects in our study. Second, we did not use the most sensitive assay for AMH measurements (picoAMH assay). However, the results of Gen II and picoAMH assays are highly correlated and can be translated using an equation (picoAMH = 0.01 + 1.69*GenII) [46, 47]. Third, some factors, including variations in AMH levels across the lifespan, the existence of overlap in AMH values between PCOS and healthy subjects with good ovarian reserve, may make universal acceptance of cut-off AMH levels for the prediction of PCOS be challenging.

## Conclusions

In conclusion, age-specific cut-off values of AMH, using well-established advanced statistical methods could elegantly assess the value of AMH in discriminating PCOS patients and may be useful as an initial assay for PCOS diagnosis.

## Supplementary Information

**Additional file 1: Supplementary Figure 1.** Box plots showing the median (Inter Quartile Range) values of serum AMH in ng/ml within PCOS phenotypes. **Supplementary Table 1.** Cutoff, PPV and NPV based on Bayesian approach with 95% CI for PCOS phenotypes.

## Data Availability

The primary data for this study is available from the authors on direct request.

## Abbreviations

AMH: Anti-Müllerian hormone
PCOS: Polycystic ovary syndrome
PPV: Positive predictive value
NPV: Negative predictive value
TLGS: Tehran Lipid and Glucose cohort Study
GAMs: Generalized additive models
OA: Oligoanovulation
HA: Hyperandrogenism
PCOM: Polycystic ovarian morphology
AFC: Antral follicle count
mFG: Modified Ferriman-Gallwey
DHEAS: Dehydroepiandrosterone sulfate
LH: Luteinizing hormone
FSH: Follicle-stimulating hormone
