# Supplementary material for: Age-specific cut-off levels of anti-Müllerian hormone can be used as diagnostic markers for polycystic ovary syndrome
Source: Reprod Biol Endocrinol. 2021 May 22;19:76. doi: 10.1186/s12958-021-00755-8 (PMC8140506; doi:10.1186/s12958-021-00755-8)
Supplement: Supplementary file 1 — Additional file 1: Supplementary Figure 1. Box plots showing the median (Inter Quartile Range) values of serum AMH in ng/ml within PCOS phenotypes. Supplementary Table 1. Cutoff, PPV and NPV based on Bayesian approach with 95% CI for PCOS phenotypes. [file 12958_2021_755_MOESM1_ESM.pdf]

Age-specific cut-off levels of anti-Müllerian hormone can be used as diagnostic markers for polycystic ovary syndrome

Journal Name: Reproductive Biology and Endocrinology

Fahimeh Ramezani Tehrani*, Maryam Rahmati, Fatemeh Mahboobifard, Faezeh Firouzi, Nazanin Hashemi, Fereidoun Azizi

*Corresponding author's e-mail and ground mail addresses, telephone and fax numbers:

Fahimeh Ramezani Tehrani, Reproductive Endocrinology Research Center, Research Institute for Endocrine Sciences 24 Parvaneh, Yaman Street, Velenjak, P.O. Box:19395-4763, Tehran, I.R. Iran. Postal Code: 1985717413

Tel: 98-21-22409309

Supplementary figure1. Box plots showing the median (Inter Quartile Range) values of serum AMH in ng/ml within PCOS phenotypes.

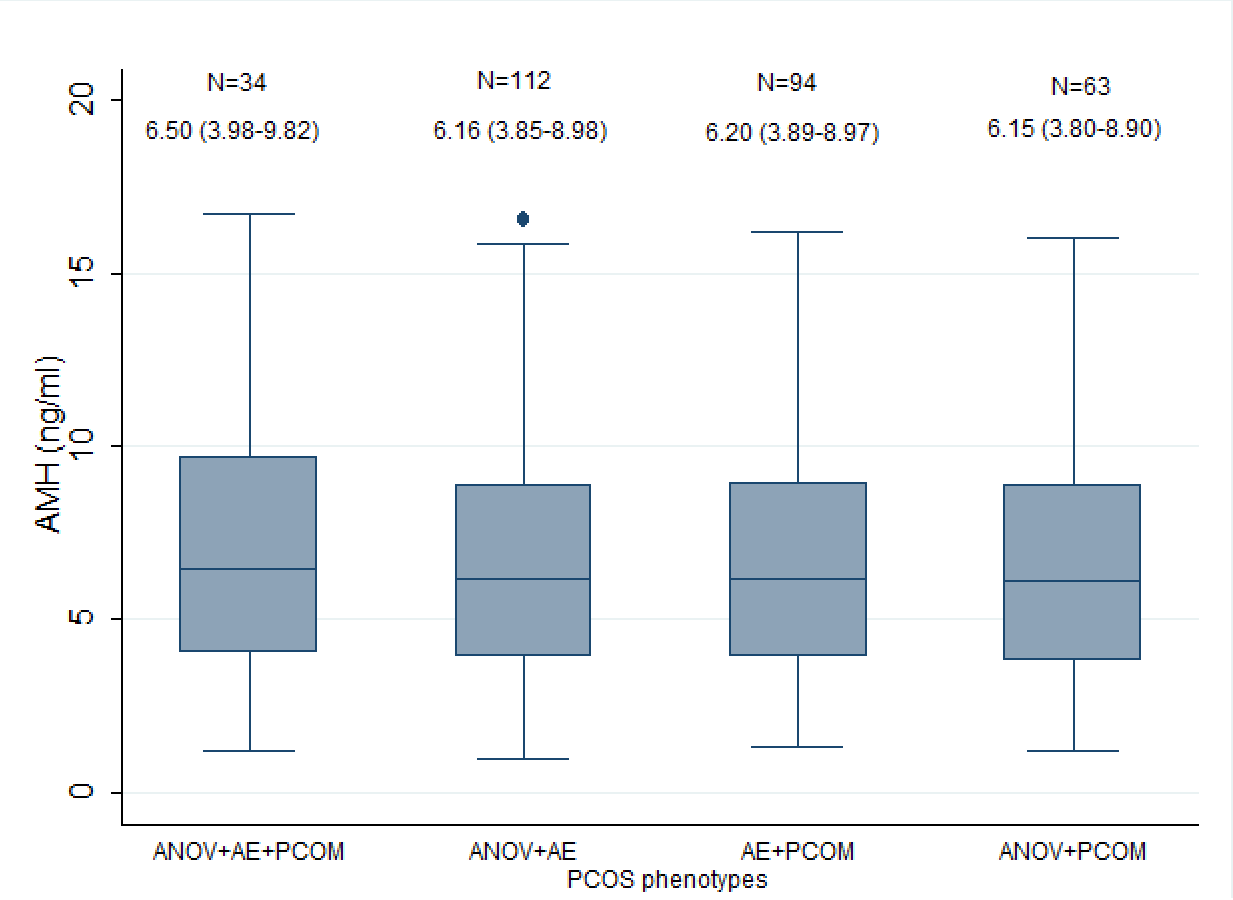

Note: ANOV= Anovulation, AE = Androgen excess, PCOM = polycystic ovarian morphology

Supplementary table 1. Cutoff, PPV and NPV based on Bayesian approach with 95% CI for PCOS phenotypes.

PCOS phenotypes	Estimated cutoff	PPV	NPV
ANOV+AE+PCOM	5.43 (5.10, 6.04)	0.91 (0.75,0.99)	0.97 (0.95,0.99)
ANOV+AE	5.19 (5.02,5.22)	0.95 (0.89,0.99)	0.92 (0.89,0.94)
AE+PCOM	5.18 (5.05,5.70)	0.95 (0.87,0.99)	0.96 (0.91,0.95)
ANOV+PCOM	5.51 (5.09, 5.64)	0.96 (0.87, 0.99)	0.83 (0.93, 0.96)

Note: PPV= Positive predictive value, NPV= Negative predictive value, CI= confidence interval.
